# Transparent planar solar absorber for winter thermal management

**DOI:** 10.1038/s41598-022-19448-2

**Published:** 2022-12-25

**Authors:** Muhammad Saad Asad, M. Z. Alam

**Affiliations:** Department of Electrical and Computer Engineering, Queen’s University, Kingston, Canada

**Keywords:** Metamaterials, Nanocavities

## Abstract

Indoor heating during winters accounts for a significant portion of energy consumed by buildings in regions of cold climate. Development of transparent coatings for windows that efficiently harvest solar energy can play a major role in reducing energy consumption and fuel costs incurred for winter heating. In recent years, there has been a great research effort towards designing transparent solar absorber coatings using nanophotonic structures. The potential of coatings based on planar multilayer structures, however, has received very little attention. In this work we investigate the performance of planar multilayer thin films using low-cost materials for design of transparent solar absorber window coatings. Our study led to the proposal of two planar multilayer designs. Simulation results predict that an increase in surface temperature by 27 K and 25 K, while maintaining mean visible transmittance of over 50% is possible using these designs. These results illustrate the great promise planar multilayer structures hold for winter thermal management of buildings.

## Introduction

Buildings are responsible for more than 30% of the energy consumption worldwide^1,2^ and 19% of the global green house gas emission^3^. Indoor heating in cold climates is a significant contributor to the energy demand of buildings. The use of fossil fuel for heating not only negatively affects the environment, but the high cost of fossil fuels also results in widespread energy poverty^4^. There is an urgent need to develop simple and inexpensive methods of passive heating to lower building energy usage and costs incurred for winter heating. Current methods of passive heating including double-glazed window, Trombe wall and solarium have several limitations. These schemes often require significant investment and cannot be easily retrofitted to existing buildings^5^. In recent years, the rapid progress of nanophotonics has provided us greatly improved control on light-matter interaction allowing new ways of harnessing solar energy for passive heating. This has led to the development of transparent solar absorber coatings based on metamaterial and metasurface architectures for passive winter thermal management ranging from applications of indoor heating^6–9^ to de-icing and antifogging applications^10–12^.

Windows are an important part of most buildings and are often responsible for more than 25% of heat loss in a typical building^13^. Unlike walls, windows need to be transparent to visible light, making it challenging to avoid heat loss through them. Furthermore, in addition to being felt cold while touched, a cold window will result in a draft, causing discomfort anywhere in the room. Thermal comfort does not only depend on air temperature, but also on factors such as air motion, relative humidity and mean radiant temperature (MRT)^6,14–19^. Shifting the MRT can significantly impact thermal comfort. MRT describes the radiant environment of an object and is the weighted average temperature of all the exposed surfaces in a given space. During cold days, increasing the MRT by 3 °C can allow the indoor air temperature to be decreased by 2 °C without affecting indoor thermal comfort^6,16^. More than 50% of the solar energy lies in the near-infrared (NIR) region, which does not contribute to illumination^13^. By designing specialized window coatings that effectively absorb NIR solar radiation to raise the surface temperature one can, in turn, raise the MRT of an indoor space. Such technology will allow the possibility of lowering the indoor air temperature without affecting thermal comfort and hence reduce both cost and energy demand for indoor heating.

Optical metamaterial and metasurface have emerged as a very promising technology for the implementation of transparent solar absorber coatings. 8 K rise in surface temperature of a glass substrate while maintaining 75% overall visible transmittance was reported using a metamaterial design based on nickel nanoantennas^6^. Application of hybrid plasmonic metasurface coating based on gold nanoparticles embedded in a TiO_2_ matrix for antifogging application was investigated in^12^ which achieved a mean visible transmittance of 36%. An asymmetric metasurface architecture based on periodic titanium/aluminum oxide/copper arrays for transparent solar absorber was reported in^8^. Numerical analysis predicted a mean visible transmittance above 60% and mean absorption of 45% for this design. Among more recent works, a planar linear gold and silver nanoparticle assembly coated on a glass substrate demonstrated temperature increase by 9.8 K while visible transparency was larger than 65%^9^. Another recent work demonstrated 38 K rise in surface temperature using a coating based on cesium (Cs) doped tungsten oxide (WO_3_) nanoparticles for antifogging applications^10^. The coating was fabricated using simple fabrication process and showed excellent stability. Unfortunately, Cs is a rare earth metal and due to its scarcity and usefulness, has been classified as a critical mineral for US national security^20^. Therefore, it will be challenging to use this design for applications where cost would be a major consideration.

Previous works on passive heating focused on either periodic structures or nanoparticles. Metal/insulator/metal (MIM) is an important class of nanophotonic structures that has been investigated for many applications including broadband solar absorption^21–25^ and selective visible and NIR filters^26^ due to its advantages e.g. ability to enhance light-matter interaction and ease of fabrication. However, the potential of planar MIM architecture for development of transparent solar energy absorber coatings has received very little attention. In this work, we carried out a systematic analysis of the potential of planar MIM structure for such an application with a focus on designs that can be implemented using low-cost materials.

The rest of the paper is organized as follows. In “Methods” section we reviewed the design geometry investigated in this work and methodology used for optical analysis. In “Results” section we carried out a detailed parametric study using a combination of transfer matrix method and particle swarm optimization (PSO) to determine the optimum choices of materials and device dimensions. We focused on designs which can be fabricated using low-cost materials and inexpensive and scalable fabrication process. We found that TiN/SiO_2_/TiN configuration provides a combination of good transmission in the visible wavelength range and good overall absorption. Further analysis revealed that addition of two thin TiO_2_ layers enhances the mean and maximum transmittance in the visible region. We then carried out thermal simulation for these two optimized designs and found that under AM 1.5 solar illumination these designs are capable of increasing the steady-state temperature of a glass surface by 27 K and 25 K respectively. These results are significantly better than many previously reported nanophotonic structures for passive heating of windows, which requires the use of noble metals or complex fabrication process steps. The “Discussion” section concludes the paper with comparison of our designs with available alternatives, and suggestions about potential applications of our designs.

## Methods

### Overview of the design process

**Figure 1:**
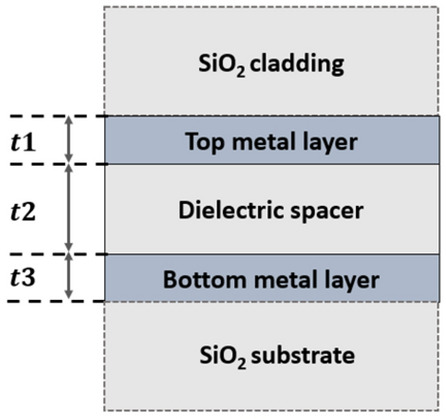
Schematic of the MIM design investigated for passive heating.

The purpose of this work is to develop a transparent solar absorber that can be fabricated using low-cost materials with simple, inexpensive, and scalable fabrication process. To achieve this, we chose to base our initial design on the MIM architecture shown in Fig. 1 because it can be fabricated using simple thin film deposition process and does not require any patterning step.

We attempted to find designs, which show high visible light transparency (400–700 nm) and high NIR absorption (700–2500 nm). As described in^24,25^, even for a simple MIM structure, the light-matter interaction can be quite complex. Therefore, utilizing a suitable optimization algorithm is required for finding the optimum solution. We use a combination of PSO and transfer matrix formulation to optimize our designs. Application of transfer matrix allows fast and accurate determination of transmission, reflection, and absorption of planar multilayer stacks^27,28^. In the following discussion we will briefly review the transfer matrix method. This discussion closely follows the description reported in^27^.

**Figure 2:**
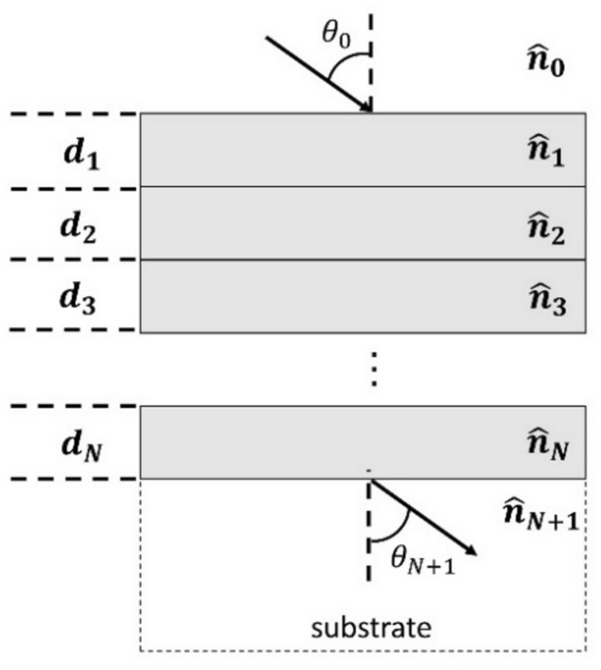
Planar multilayer structure.

Fig. 2 shows a multilayer structure consisting of $N$ layers of finite thicknesses sandwiched between a cladding (layer 0) and a substrate (layer $N + 1$). The thickness of the $ith$ layer is given by $d_{i}$ and its complex refractive index is $\hat{n}_{i} = n_{i} + jk_{i}$.

$\theta_{i}$ denotes the angle at which light propagates in medium $i$ with respect to the normal at the interface of medium $i - 1$ and $i$. The transfer matrix relating the electric field entering and leaving the stack is given by

1 $$M = \left( {\begin{array}{*{20}c} {M_{11} } & {M_{12} } \\ {M_{21} } & {M_{22} } \\ \end{array} } \right) = D_{0,1} P_{1} D_{1,2} P_{2} D_{2,3} P_{3} \ldots P_{N} D_{N,N + 1}$$

here $D_{i,i + 1}$ is the matrix relating the forward and backward propagating electric field quantities at the two sides of the interface between layer $i$ and layer $i + 1 \left( {i = 0,1,..,N} \right)$.

2 $$D_{i,i + 1} = \frac{1}{{t_{i,i + 1} }}\left[ {\begin{array}{*{20}c} 1 & {r_{i,i + 1} } \\ {r_{i,i + 1} } & 1 \\ \end{array} } \right]$$

Here $r_{i,i + 1}$ and $t_{i,i + 1}$ are Fresnel reflection and transmission coefficients between layer $i$ and $i + 1$. For a TE or s polarized incident wave, they are given by

3 $$r_{i,i + 1} = \frac{{\hat{n}_{i} cos\theta_{i} - \hat{n}_{i + 1} cos\theta_{i + 1} }}{{\hat{n}_{i} cos\theta_{i} + \hat{n}_{i + 1} cos\theta_{i + 1} }}$$

4 $$t_{i,i + 1} = \frac{{2\hat{n}_{i} cos\theta_{i} }}{{\hat{n}_{i} cos\theta_{i} + \hat{n}_{i + 1} cos\theta_{i + 1} }}$$

For a TM or p polarized incident wave, these quantities are given by

5 $$r_{i,i + 1} = \frac{{\hat{n}_{i} cos\theta_{i + 1} - \hat{n}_{i + 1} cos\theta_{i} }}{{\hat{n}_{i} cos\theta_{i + 1} + \hat{n}_{i + 1} cos\theta_{i} }}$$

6 $$t_{i,i + 1} = \frac{{2\hat{n}_{i} cos\theta_{i} }}{{\hat{n}_{i} cos\theta_{i + 1} + \hat{n}_{i + 1} cos\theta_{i} }}.$$

The field quantities at the top and bottom interfaces of the $ith$ layer are related by the propagation matrix $P_{i} \left( {i = 1, \ldots ,N} \right)$ which is given by

7 $$P_{i} = \left[ {\begin{array}{*{20}c} {e^{{ - j\varphi_{i} }} } & 0 \\ 0 & {e^{{ + j\varphi_{i} }} } \\ \end{array} } \right]$$

where $\varphi_{i}$ is the phase shift experienced by the wave when it propagates a distance $d_{i}$ through the $ith$ layer, and has the following expression

8 $$\varphi_{i} = \frac{2\pi }{\lambda }\hat{n}_{i} d_{i} cos\theta_{i}$$

Once the transfer matrix $M$ is calculated, the reflection $R$ and transmission $T$ of the multilayer stack can be found by using the following expressions,

9 $$R = \left| {\frac{{M_{21} }}{{M_{11} }}} \right|^{2}$$

10 $$T = \frac{{n_{N + 1} cos\theta_{N + 1} }}{{n_{0} cos\theta_{0} }}\left| {\frac{1}{{M_{11} }}} \right|^{2}$$

where $n_{N + 1}$ is the real part of the refractive index of the substrate and $n_{0}$ is the real part of the refractive index of the cladding.

The absorption of the multilayer stack can be determined using the relation

11 $$A\left( \lambda \right) = 1 - R\left( \lambda \right) - T\left( \lambda \right).$$

To optimize our designs, we chose to use PSO due to its ability to find an optimum solution from a very large solution space without the need to make assumptions about the nature of the optimization problem^29–33^. In PSO, the optimization process starts with several trial solutions, which are called particles. After each iteration, each particle updates its local optimal solution found near its vicinity as well as the global optimal solution found because of the movement of all particles through the search space. We carried out the optimization in two steps. In step 1, we investigated the simple MIM architecture sandwiched between SiO_2_ substrate and cladding. This analysis allowed us to identify the optimal choice of materials and layer thicknesses for the MIM design. In Step 2, we extended this analysis and added additional TiO_2_ layers on top and bottom of the optimized MIM structure and re-optimized the layer thicknesses.

### Heat transfer modelling

To determine the rise in temperature for our proposed designs, the thermal simulation needs to take into consideration different mechanisms of heat transfer. Here we briefly discuss these processes.

The temperature variation through thermal conduction can be modelled using the differential heat transport equation

12 $$Q = \rho c_{p} \frac{\partial T}{{\partial t}} - \nabla \cdot \left( {k\nabla T} \right)$$

where $Q$ is the heat source or absorbed energy (W), $\rho$ is the density of the material (kg‧m^−3^), $c_{p}$ is the specific heat capacity (J‧Kg^−1^‧K^−1^), $T$ is the temperature of the object (K), $t$ is time (seconds), and $k$ is the thermal conductivity (W‧m^−1^‧K^−1^).

At thermal equilibrium, the net heat absorbed is equal to the net heat dissipated. The heat transport equation under this condition simplifies to the following

13 $$Q = - \nabla \cdot \left( {k\nabla T} \right).$$

Since the coating surface is in contact with air, heat loss from the surface will occur through convection. Heat loss through convection $\left( {Q_{conv} } \right)$ is directly proportional to the temperature difference between coating surface and its surroundings and can be modelled using

14 $$Q_{conv} = Ah_{c} \left( {T - T_{0} } \right)$$

where $A$ is the surface area of the object (m^2^), $h_{c}$ is the convective heat transfer coefficient (W‧m^−2^‧K^−1^), $T$ is the temperature of the object and $T_{0}$ is the temperature of ambient air (K).

Heat removed from the coating surface in the form of long wave radiation ($Q_{rad}$) back to surrounding air is described as

15 $$\frac{{Q_{rad} }}{A} = \varepsilon \sigma \left( {T^{4} - T_{0}^{4} } \right)$$

where $\varepsilon$ is the emissivity of the object (dimensionless), and $\sigma$ is the Stefan-Boltzmann constant (W‧m^−2^‧K^−4^).

## Results

### Determination of optimal material choice and layer thicknesses

To determine the optimal material choice and layer thickness of the MIM design, we first investigated different plasmonic materials for the top and bottom layers. The materials investigated included Al, Cu, Ni, TiN and Au. Al has been extensively studied due to its strong plasmon response in the visible region^34,35^. Cu and Ni are low-cost metals and have been studied by other groups for transparent thermal management applications^6–8,36^. Transition metal nitride TiN is considered a good candidate due to its low-cost and higher mechanical and thermal stability than noble metals^37,38^. We included Au in our investigation to determine the possible compromise in performance that we may have to accept when we use cheaper plasmonic materials. Optical properties of Cu, Al and Au were taken from^39^, and those for Ni were taken^40^. For TiN, the refractive index data was obtained from^40,41^. The dielectric spacer region separating the metallic layers for this analysis is assumed to be SiO_2_. Optical properties of SiO_2_ were taken from^40^. Effect of the choosing other spacer materials will be analyzed later in the paper.

As stated in “Methods” section, since our designs are based on planar multilayer MIM stacks, transfer matrix formulation together with PSO was used to simultaneously maximize both visible transmittance and NIR absorption. Many previous reports on transparent coatings for passive heating presented results for normal incidence. Although the transparent coating under discussion can be useful for various applications, the most likely application would be as a coating for windows of buildings for passive heating. Sunlight will always be incident at an inclined angle on the window in such a case. We decided to optimize our design for 45° angle of incidence. We also carried out performance analysis for various angles of incidence for the optimized designs. The transfer matrix method was implemented using MATLAB. The accuracy of this code was verified by comparing the results obtained from this code with those obtained from Lumerical STACK from Ansys. The optimization procedure was implemented using the PSO toolbox provided by MATLAB.

For the optimization procedure, we aimed to maximize two objective functions: the mean visible transmittance and the mean NIR absorption. In order to simultaneously maximize both quantities we utilized the conventional weighted aggregation (CWA) approach for multi-objective optimization^32,33^. In this method, all objective functions are summed to form one single objective function that the optimization algorithm aims to either maximize or minimize. Each objective function is associated with a non-negative weight that remains fixed during the optimization. We used the following figure of merit (FOM) to optimize our planar multilayer designs.

16 $$FOM = w_{1} mean\left( {T_{vis} } \right) + w_{2} mean\left( {A_{NIR} } \right)$$

where $T_{vis}$ is the visible transmittance, $A_{NIR}$ is the absorption in the NIR region and $w_{1}$ and $w_{2}$ are non-negative weights such that

17 $$w_{1} + w_{2} = 1$$

For the optimization, we used 15 particles per iteration and carried out 30 iterations, running a total of 450 simulations for each material configuration. The layer thicknesses were set as the design parameter. The upper and lower limits set for the thickness parameter were 2 nm and 20 nm for the top and bottom layers and 2 nm and 500 nm for the middle SiO_2_ dielectric spacer.

The layer thicknesses and the optical performance of the optimized designs are summarized in Table 1. The last column of Table 1 gives the solar power absorbed by each design under AM 1.5 illumination. This data was obtained by first multiplying the normalized absorption profile with the solar spectrum (AM 1.5), followed by integrating the resulting spectra over the 280 nm to 2500 nm wavelength range to calculate the absorbed power density.

**Table 1:** Summary of layer thicknesses and optical performance for the optimized MIM design for various choices of metals. The spacer is SiO_2_.

*t*1 (nm)	*t*2 (nm)	*t*3 (nm)	Metal/metal nitride	Mean transmittance in visible	Maximum transmittance in visible	Absorbed solar power density (W/m^2^)
2	254	4	Al	0.269	0.336	434.6
4	500	9	Au	0.601	0.837	160.9
2	500	4	Cu	0.706	0.894	185.1
3	214	4	Ni	0.468	0.490	557.2
4	210	4	Ni	0.426	0.452	583.2
5	228	8	TiN	0.518	0.566	573.6

**Figure 3:**
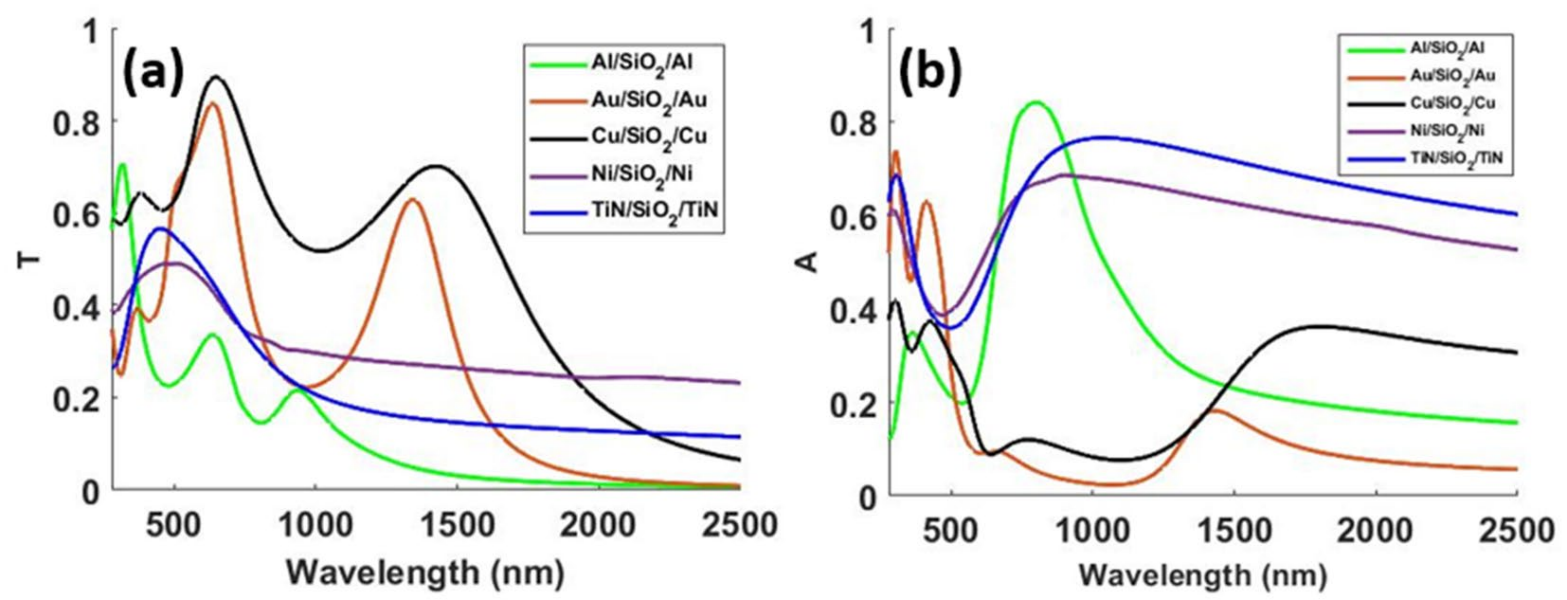
(**a**) Transmittance ‘T’ and (**b**) absorption ‘A’ spectra for the optimized designs for different choices of top and bottom materials.

Fig. 3 shows transmittance and absorption profiles obtained for the optimized designs presented.

**Figure 4:**
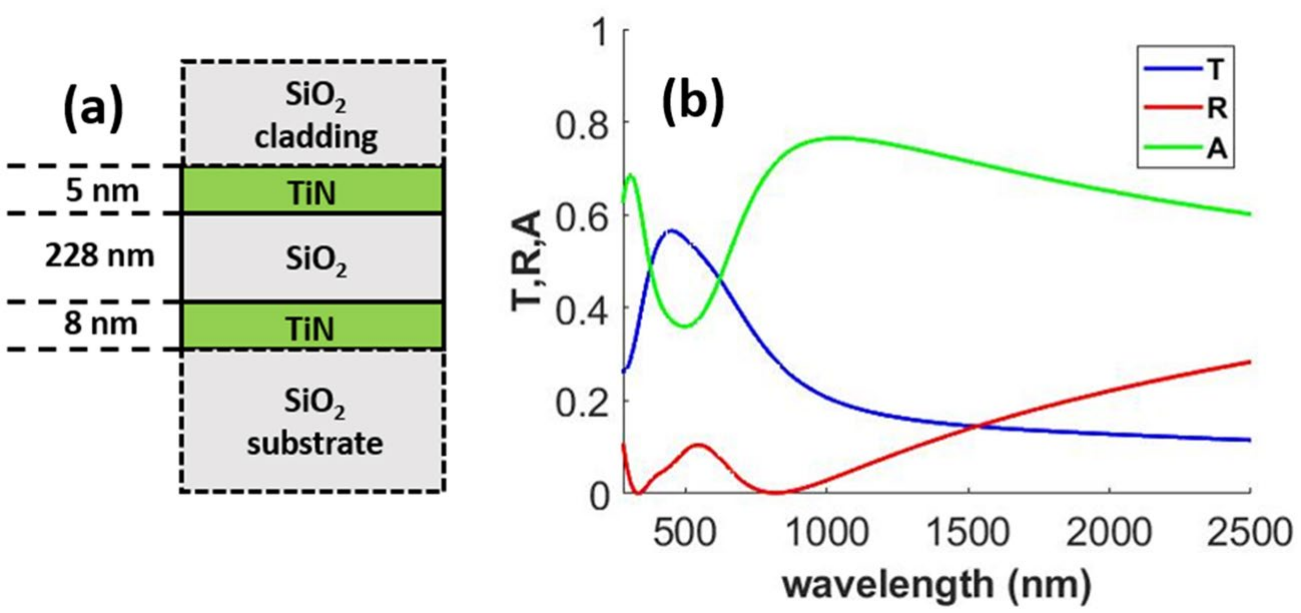
(**a**) Schematic and (**b**) optical response of the optimized TiN/SiO_2_/TiN MIM design.

Au has been extensively used in the past for transparent passive heating applications^6,8,9,11,12^. However, our study reveals that cheaper alternatives perform much better for applications requiring high solar absorption in case of the MIM geometry. The TiN and Ni-based designs provide significantly better trade-off between mean transmittance in the visible region and absorbed solar power density compared to the other designs. In Table 1, we present two Ni-based designs. The first Ni-based design with top metal layer thickness $t1 = 3 {\text{ nm}}$, was a result of our optimization. Depositing 3 nm Ni film is however a challenging task. Deposition of uniform Ni film of thickness 4–7 nm using simple physical vapor deposition process has been reported in literature^42^. We therefore present a second Ni design after increasing the minimum thickness of the top metal layer to 4 nm. Increasing the top metal layer thickness showed that good performance in terms of higher solar power absorption can be achieved at the cost of lower mean visible transmittance. Although the 2nd Ni-based design presented shows higher solar absorption than the TiN-based design, the TiN-based design shows significantly higher mean transmission compared to both Ni designs. Therefore, we chose the TiN-based design for further investigation. Although the optical response of this design was presented in Fig. 3 with those for other designs, we reproduce the optical response for the TiN-based design in Fig. 4 for ease of visualization.

The analysis presented so far assumed that the spacer material separating the metallic layers is SiO_2_. We now consider two alternate choices of spacer material: aluminum oxide (Al_2_O_3_) and silicon nitride (SiN). Refractive index data for Al_2_O_3_ and SiN are taken from^40^ and^43^ respectively. We reoptimized our designs for these new choices of spacer materials. Table 2 summarizes the optimized designs and key performance results for the three choices of spacer material.

**Table 2:** Summary of layer thicknesses and optical performance for the optimized MIM design for various choices of spacer materials. The metallic layers are TiN for all the designs.

*t*1 (nm)	*t2* (nm)	*t*3 (nm)	Dielectric	Mean transmittance in visible	Maximum transmittance in visible	Absorbed solar power density (W/m^2^)
5	228	8	SiO_2_	0.518	0.566	573.6
4	155	12	Al_2_O_3_	0.449	0.511	566.0
3	120	12	SiN	0.452	0.529	518.3

**Figure 5:**
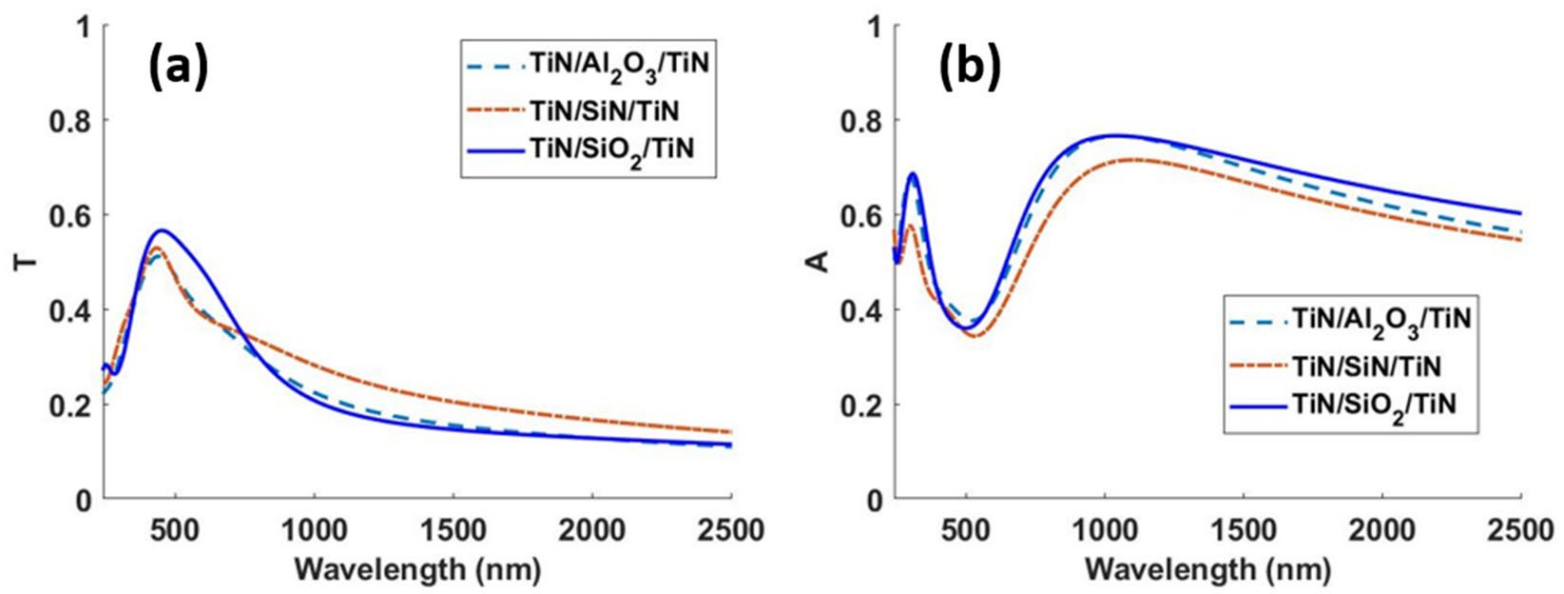
(**a**) Transmittance ‘T’ and (**b**) absorption ‘A’ spectra for the optimized designs for various choices of spacer materials. The plasmonic material is TiN for all the designs.

The optical response for these three designs is presented in Fig. 5. Among the three choices for spacer materials we considered, the SiO_2_ design shows the highest mean visible transmittance and solar power absorption. Therefore, we chose SiO_2_ as the spacer medium for the rest of our paper.

### Effect of adding additional TiO_2_ layers to the TiN/SiO_2_/TiN MIM design

**Figure 6:**
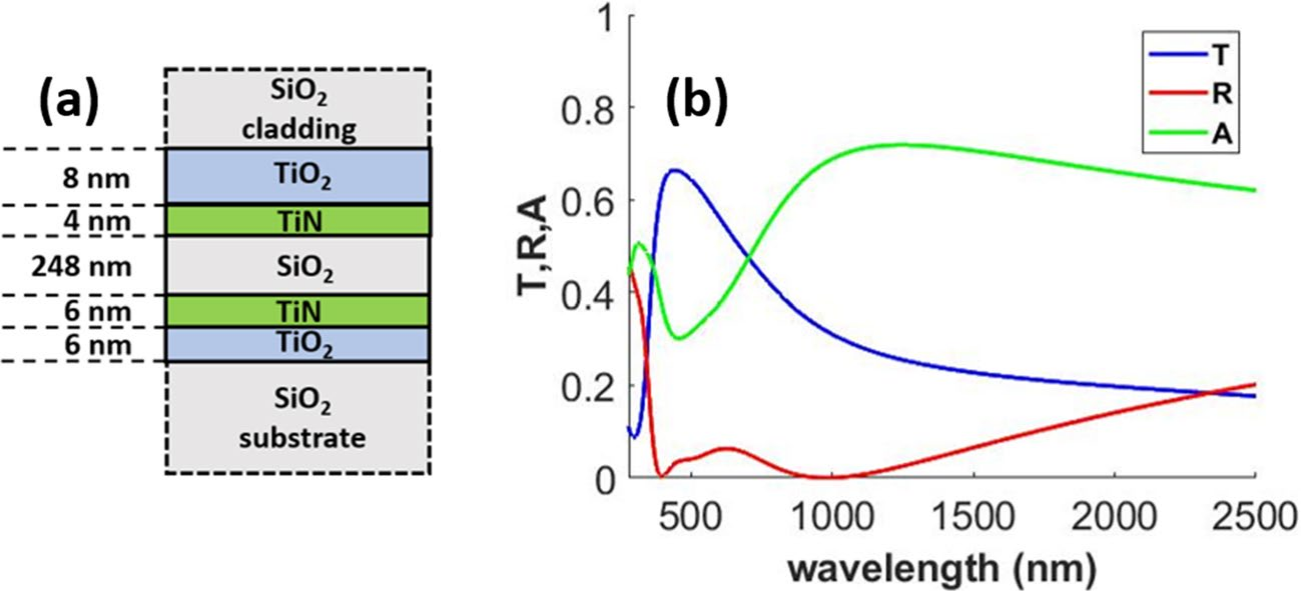
(**a**) Proposed TiO_2_/TiN/SiO_2_/TiN/TiO_2_ design based on insulator/MIM/insulator architecture and (**b**) optical response the optimized design.

Our choice of MIM geometry for passive solar heating was motivated by previous reports on the applications of this geometry for the design of perfect absorbers^21–25^. The inverse of this geometry (insulator/metal/insulator or IMI), on the other hand has been used for enhancing transmission^36,44–47^. We now investigate a combination of MIM and IMI structures to see if such a combination can provide superior performance than what is achievable from MIM alone. As shown in Fig. 6a, the new structure is achieved by adding TiO_2_ layers on the two sides of the TiN/SiO_2_/TiN structure analyzed in “Determination of optimal material choice and layer thicknesses” section. TiO_2_ has a higher refractive index than SiO_2_ and has high mechanical strength, which makes it a suitable choice for our design^36,46,47^.

As done in “Determination of optimal material choice and layer thicknesses” section, transfer matrix formulation in conjunction with PSO is utilized to optimize the layer thicknesses. The upper and lower limits set for the thickness parameter were 2 nm and 50 nm for the TiN layer and 2 nm and 500 nm for the dielectric layers. Addition of two TiO_2_ layers increased the complexity of optimization, therefore the total number of particles used per iteration was increased to 30. 40 iterations were carried out, running a total of 1200 simulations. The optimized design and its optical response are shown in Fig. 6a,b respectively.

A comparison of the optical response before and after addition of the TiO_2_ layers to the TiN/SiO_2_/TiN design is presented in Table 3. We designate the TiN/SiO_2_/TiN and the TiO_2_/TiN/SiO_2_/TiN/TiO_2_ designs as Design 1 and Design 2 respectively. Although the addition of TiO_2_ layers causes a reduction in solar absorption, it significantly enhances both the mean and the maximum visible transmittance.

**Table 3:** Summary of optical and thermal performance results Design 1 and Design 2.

Multilayer design	Mean transmittance in visible	Maximum transmittance in visible	Absorbed solar power density (W/m^2^)	Simulated temperature increase (K)
Design 1	0.518	0.566	573.6	27
Design 2	0.611	0.664	505.2	25

### Thermal simulations

**Figure 7:**
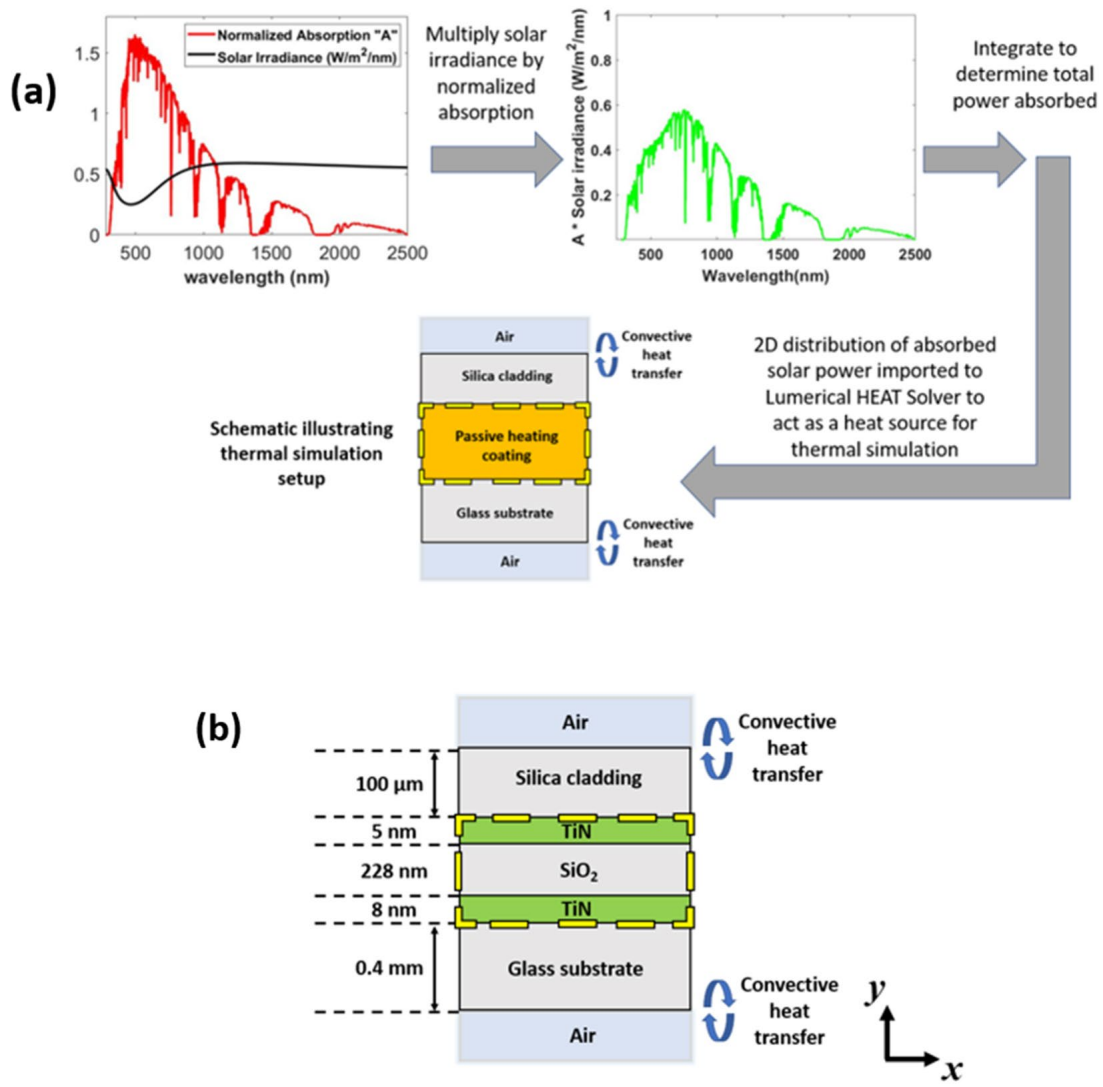
(**a**) Thermal simulation process flow and (**b**) thermal simulation setup for Design 1.

In “Determination of optimal material choice and layer thicknesses” and “Effect of adding additional TiO_2_ layers to the TiN/SiO_2_/TiN MIM design” sections we have identified two designs which provide good transmission in the visible region, while absorbing a significant amount of solar energy. In this section, we examine the temperature rise achievable when the proposed structures are coated on a glass substrate. The results presented here are obtained using a combination of Lumerical FDTD and Lumerical HEAT Solver from Ansys. The process flow showing the procedure carried out to determine thermal performance of the of our designs is represented in Fig. 7a. A schematic of the simulation setup for Design 1 is shown in Fig. 7b.

We first carried out 2D FDTD simulations to determine the spatial absorption under normal illumination which is then multiplied by the solar spectrum (AM 1.5) and integrated over the 280 nm to 2500 nm wavelength range to determine the spatial distribution of absorbed power in the xy plane over the area bounded by the dashed yellow lines in Fig. 7a. This absorption data was then exported to HEAT. For thermal simulation in HEAT, the x-span was set to 0.4 µm similar to the one used for FDTD simulation, and the planar multilayer structure was placed on an 0.4 mm thick SiO_2_ substrate. A 100 µm SiO_2_ coating was applied to the top of the MIM structure to act as a cladding. The ambient temperature of the simulation was kept at 295 K. We would like to mention that to evaluate the reliability of our thermal simulations, we have reproduced results from several previous reports. Details for some of these are provided in the Supplementary Information.

Convective surface boundaries depicted in Fig. 7b are applied to the top and bottom of the simulation region to simulate heat dissipation from the device. The heat transfer coefficient for these boundaries was set to 10 Wm^−2^ K^−1^ which is a common value used for air and the atmospheric temperature surrounding the device is assumed to be uniform at 295 K. The effect of varying the boundary conditions is discussed in the Supplementary Information. A similar simulation setup was also used for Design 2. The thermal simulations predicted that under AM 1.5 solar illumination, the steady state temperature of the glass substrate will rise by 27 K for Design 1 and 25 K for Design 2.

**Figure 8:**
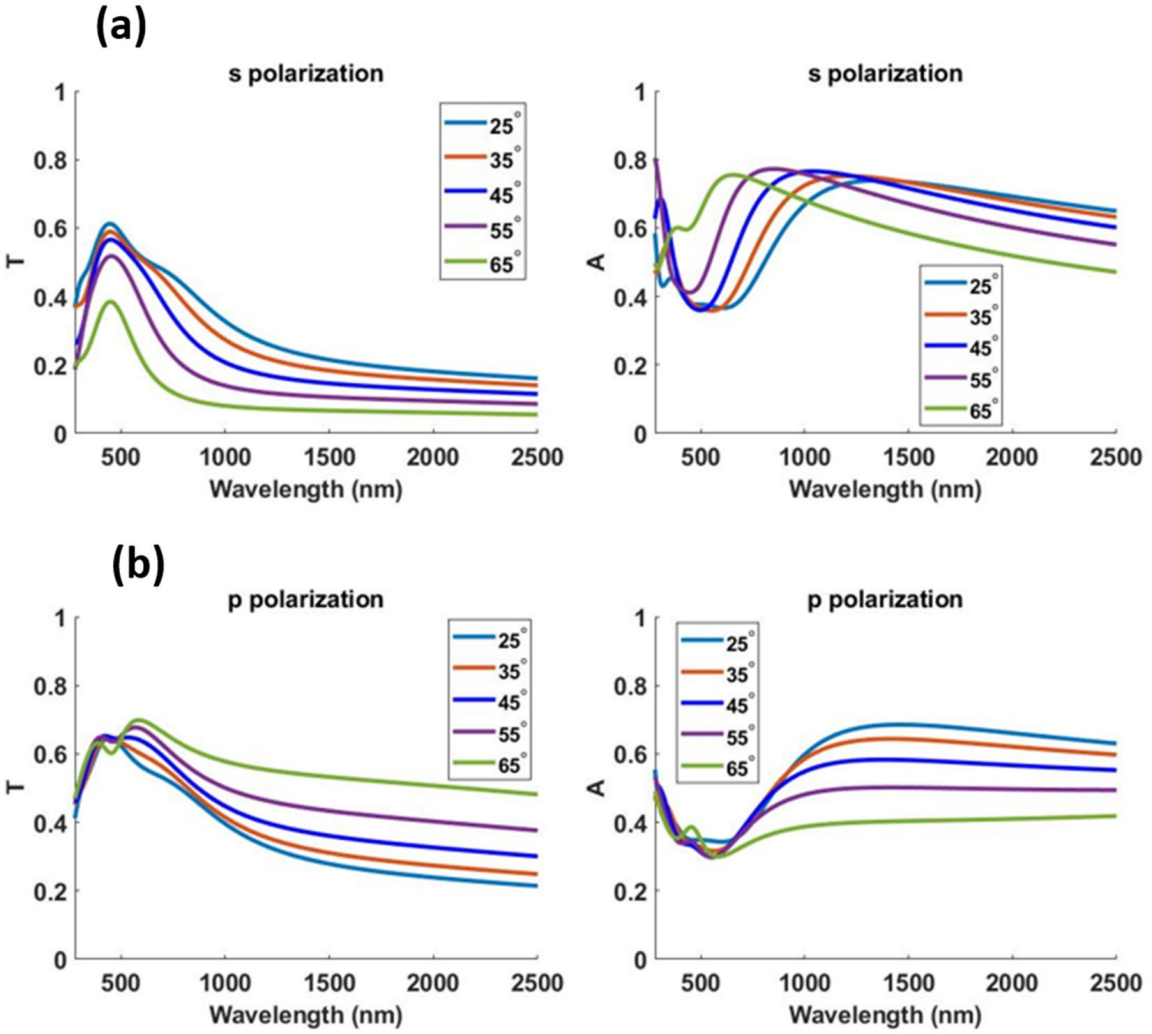
(**a**) Transmittance ‘T’ and (**b**) absorption ‘A’ profile for Design 1 at 25°, 35°, 45°, 55° and 65° angle of incidence using s and p polarized light.

**Figure 9:**
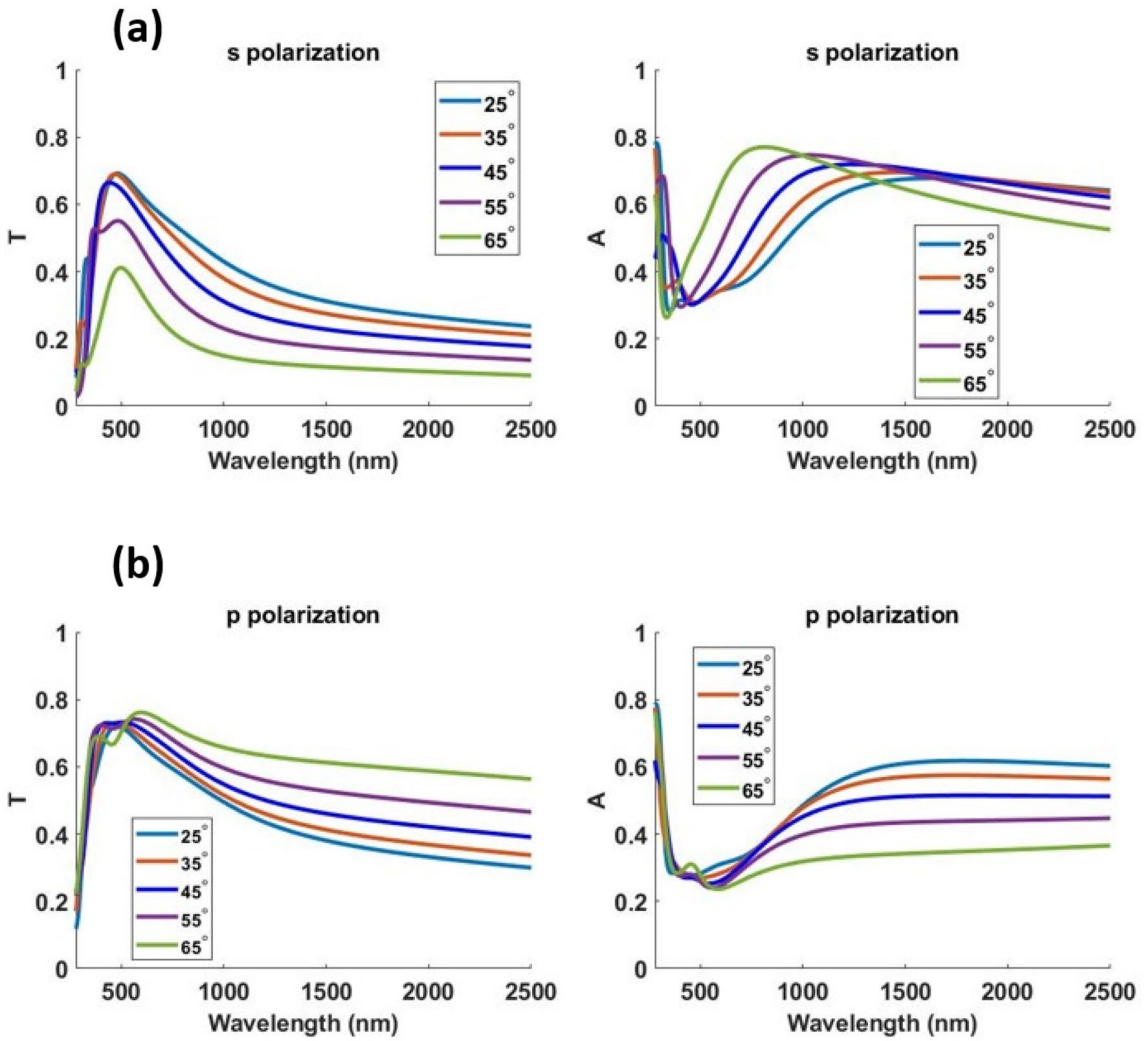
(**a**) Transmittance ‘T’ and (**b**) absorption ‘A’ profile for Design 2 at 25°, 35°, 45°, 55° and 65° angle of incidence using s and p polarized light.

For our analysis thus far, we used a linear (s polarized) plane wave source incident at an angle of 45° to the device surface. Now we extend our analysis to p polarization and also to unpolarized light since sunlight by nature is unpolarized. Results for unpolarized light source can be obtained by adding the results of the two orthogonal polarizations incoherently using the equation

18 $$\langle\left| E \right|^{2}\rangle = \frac{1}{2}\left| {\vec{E}_{s} } \right|^{2} + \frac{1}{2}\left| {\vec{E}_{p} } \right|^{2}$$

where $\left| E \right|$ is the amplitude of the electric field for an unpolarized light source and $\left| {\vec{E}_{s} } \right|$ and $\left| {\vec{E}_{p} } \right|$ are the amplitude of the electric field for s and p polarizations. Fig. 8 and Fig. 9 show the optical performance of Design 1 and 2 respectively for s and p polarization at different angles of incidence.

Tables 4 and 5 summarize the optical and thermal results obtained when simulating an unpolarized light source at different angles of incidence for Design 1 and 2 respectively.

**Table 4:** Optical and thermal performance of Design 1 for an unpolarized solar light source.

Angle of incidence (degrees)	Mean transmittance in visible	Maximum transmittance in visible	Absorbed solar power density (W/m^2^)	Simulated temperature increase (K)
25	0.583	0.629	478.6	23
35	0.583	0.619	487.5	24
45	0.579	0.607	499.5	25
55	0.551	0.578	512.0	25
65	0.476	0.496	511.8	25

**Table 5:** Optical and thermal performance of Design 2 for an unpolarized solar light source.

Angle of incidence (degrees)	Mean transmittance in visible	Maximum transmittance in visible	Absorbed solar power density (W/m^2^)	Simulated temperature increase (K)
25	0.664	0.706	410.1	20
35	0.672	0.709	417.6	21
45	0.666	0.697	430.2	21
55	0.615	0.637	446.6	22
65	0.532	0.568	462.0	23

The proposed designs maintain good optical and thermal performance at various source angles; a feature necessary for practical passive heating applications. As discussed in detail in the next section, these designs significantly outperform many previous transparent solar absorber designs.

## Discussion

Application of nanophotonics for passive heating of buildings can play a significant role in the reduction of global greenhouse gas emission and energy poverty. In this work we studied the potential of planar multilayer structures using low-cost plasmonic materials for this application. Our study led to the proposal of two planar multilayer designs. Design 1 is a TiN/SiO_2_/TiN MIM design, and Design 2 is a TiO_2_/TiN/SiO_2_/TiN/TiO_2_ design, which is a combination of MIM and IMI architectures. Both designs can be implemented using low-cost materials and simple, inexpensive, and scalable fabrication processes. The designs proposed outperform alternatives that require more expensive materials or complicated fabrication process steps. For example, both Design 1 and 2 are predicted to provide temperature rise which is more than double than that reported in^6^. Fabrication of the structure reported in^6^ requires multiple steps including deposition, etching and lift off. In contrast, fabrication of our proposed designs will require only deposition of multiple layers of thin films. Another very recent work proposed an asymmetric metasurface design which predicted a mean visible transmittance of above 60% and mean absorption of 45%^8^. Similar to the design reported in^6^, it also requires metal deposition, etching and lift off for its fabrication. Although the mean visible transmittance for Design 1 is lower than that reported in^8^, the mean absorption for Design 1 (calculated but not shown in Table 1) is more than 11% higher. Design 2 exhibits 3% higher mean absorption than that reported in^8^ while demonstrating similar mean transmittance.

The proposed nanophotonic designs for passive heating can be adapted to windows for buildings in a number of ways. They can be deposited directly on a glass window or on a transparent polymer coating, which can be attached to windows to achieve passive heating only during winter. It is also possible to integrate these multiplayer structures with commercially available low-emissivity (low-E) window coating technology^13^. Such a window will be able to simultaneously absorb solar energy for passive heating and prevent escape of heat from indoor by reflection. The benefit of such a technology for reduction in energy consumption would be significant for modern residential and commercial buildings, where windows form a large part of building surface. We believe the low cost, ease of fabrication and ability to provide large temperature increase make our designs attractive choices for passive heating of buildings and will encourage further exploration of planar multilayer structures for passive heating applications.

## Supplementary Information

Supplementary Information.

## Abbreviations

CWA: Conventional weighted aggregation
FDTD: Finite difference time domain
FOM: Figure of merit
HEAT: Heat transport solver
Low-E: Low emissivity
NIR: Near infrared
MIM: Metal/insulator/metal
PSO: Particle swarm optimization
