# Supplementary material for: Transparent planar solar absorber for winter thermal management
Source: Sci Rep. 2022 Dec 25;12:22325. doi: 10.1038/s41598-022-19448-2 (PMC9790891; doi:10.1038/s41598-022-19448-2)
Supplement: Supplementary file 1 — Supplementary Information. [file 41598_2022_19448_MOESM1_ESM.docx]

**Supplementary Information**

**Transparent planar solar absorber for winter thermal management**

Muhammad Saad Asad**^1,*^**

M. Z. Alam **^1^**

**^1^**Department of Electrical and Computer Engineering, Queen’s University, Kingston, Canada

^*^Corresponding author. Email: 19msa4@queensu.ca

**1. Reproduction of results reported in the literature**

The optical analysis for our work is performed by transfer matrix method is relatively straightforward. The thermal analysis is relatively more complex. To verify that our thermal simulations are done correctly, we have reproduced results from previous reports on solar absorbers. Here we have presented results for reproduction of two such publications.

In [S1], three different configurations for plasmonic nanoantenna based passive heating were investigated. Configuration 1 consists of a single layer of Au and a single layer of SiO_2_, whereas, Configuration 2 consists of three layers of Au and three layers of SiO_2_. Configuration 3 consists of elliptical nanoantenna arrays, where each element is made up of three layers of Ni and three layers of Al_2_O_3_. Table S1 shows the numerical and experimental temperature rise achieved by these designs as reported in [S1] as well as temperature rise predicted by our thermal simulation.

**Table S1.** Comparison of device temperature rise data

| Nanoantenna Design | Simulation results reported in [S1] | Experimental  results reported in [S1] | Our Simulation |
| --- | --- | --- | --- |
| Configuration 1: single-element Au | 1.8 | 2 | 2 |
| Configuration 2: three-element Au | 4 | 4 | 4 |
| Configuration 3: three-element Ni | 8 | 8 | 7.5 |

Our simulation model was also tested on planar multilayer photonic structure as reported in [S2]. [S2] reports experimental temperature rise achieved under exposure to sunlight for photonic structures based on TiO_2_, SiO_2_ and Cr. We simulated the ‘hot’ photonic structure reported in [S2]. The simulated temperature we obtained is 74.85℃. The experimental temperature for the ‘hot’ structure reported by [S2] is above 70℃ at noon (Figure 4c of reference [S2]).

**2. Performance of our designs under normal incidence**

The most likely application of the transparent solar absorber we designed is as a coating for providing passive heating of windows in buildings. The solar radiation will always be incident at an inclined angle on such windows. We take this fact into consideration and in our paper, the designs we presented were optimized for the case when light was incident at a 45°. Many recent works in literature [S1,S3,S4], on the other hand, reported performance of their designs for normal light incidence. To allow better comparison with existing literature, we reoptimized our designs for normal incidence. The optical and thermal performance of these reoptimized designs are summarized in Table S2.

**Table S2.** Summary of optical and thermal performance results for Design 1 and Design 2 (reoptimized for normal incidence)

| Multilayer design | Mean transmittance in visible (400-700 nm) | Maximum transmittance in visible and its location (400-700 nm) | Absorbed solar power density  (W/m^2^) | Simulated temperature increase  (K) |
| --- | --- | --- | --- | --- |
| Design 1 | 0.686 | 0.725 (454 nm) | 421.3 | 21 |
| Design 2 | 0.605 | 0.803 (459 nm) | 508.5 | 25 |

Figure S1 shows the layer thickness and transmittance and absorption profiles for these designs.

| 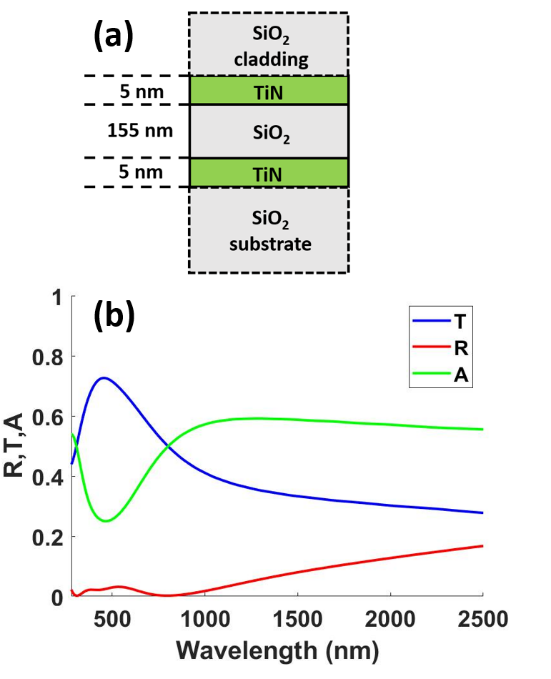 | 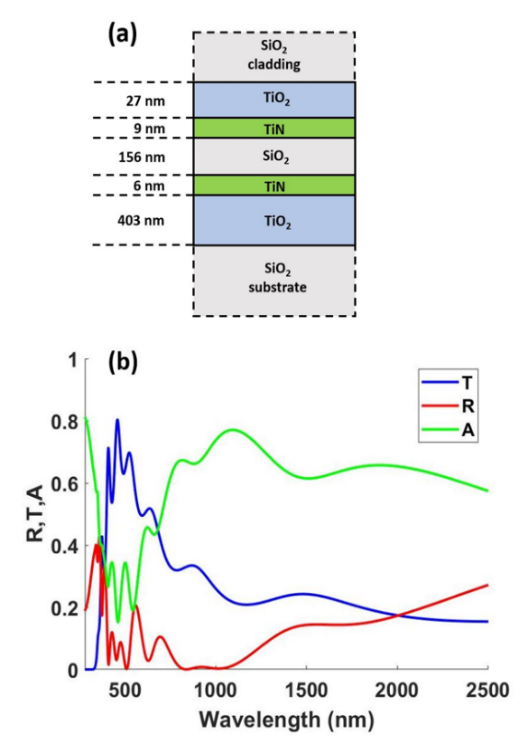 **(d)**  **(c)** |
| --- | --- |
| **Figure S1.** (a) Schematic and (b) optical response of Design 1 and (c) Schematic and (d) optical response of Design 2. | |

The designs outperform previously reported alternatives that require more expensive materials or complicated fabrication process steps. For example, the predicted temperature rises for both Designs 1 and 2 are more than double than that reported in [S1]. Another recent work which proposed an asymmetric metasurface design predicted a mean visible transmittance of above 60% and mean absorption of 45% [S4]. The mean absorption for Design 1 was calculated to be 3% lower than that reported in [S4] but the mean visible transmittance is 8% higher. Design 2 exhibits 7% higher mean absorption than that reported in [S4] while demonstrating similar mean transmittance.

**3. Effect of varying thermal boundary conditions**

The designs analyzed in this work consists of transparent coatings placed on a glass substrate, which is exposed to air on both sides. Since thermal convection will be significant for such configurations, we chose convective boundary for thermal analysis of these designs. However, there are interesting configurations that one can explore. Design of transparent thermal insulator has been reported by various groups in the past [S5]. As shown for Design 1 in Figure S2, we can consider placing the structure on such a thermal insulating surface instead of exposing it to air at the bottom surface. We have carried out additional thermal analysis for both Designs 1 and 2 when the convection boundary at the bottom is replaced by a thermal insulator boundary. Table S3 summarizes results from this analysis. Since heat cannot dissipate through the bottom surface, considerably higher temperature rise can be achieved in this case compared to the case when convection boundary is used.

| 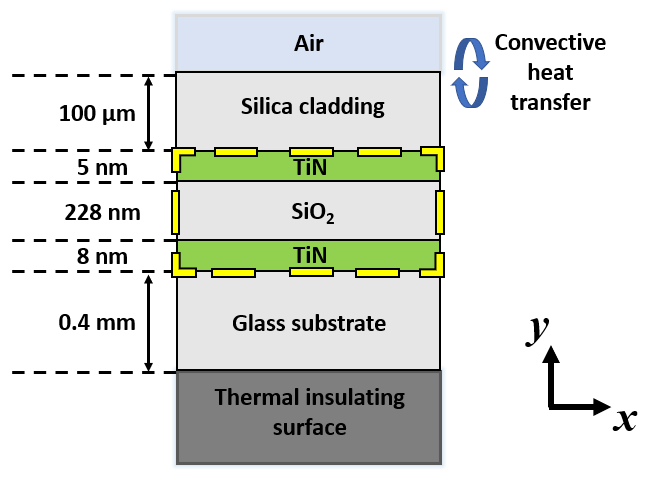 |
| --- |
| **Figure S2.** Thermal simulation setup for Design 1 placed on a thermal insulating surface. |

**Table S3.** Summary of thermal performance results for Design 1 and Design 2 for different surface boundary conditions.

| Multilayer design | Simulated temperature rise for device placed on insulating surface  (K) | Simulated temperature rise using convective boundaries for both top and bottom surfaces  (K) |
| --- | --- | --- |
| Design 1 | 58 | 27 |
| Design 2 | 51 | 25 |

**References**

[S1] Jönsson, G., Tordera, D., Pakizeh, T., Jaysankar, M., Miljkovic, V., Tong, L., Jonsson, M.P. & Dmitriev, M*.* Solar transparent radiators by optical nanoantennas. *Nano Lett.* **17**, 6766–6772 (2017).

[S2] Li, W., Shi, Y., Chen, Z. & Fan, S. Photonic thermal management of coloured objects. *Nat Commun* **9,**4240 (2018).

[S3] Ma, R., Wu, D., Liu, Y., Ye, H. & Sutherland, D. Copper plasmonic metamaterial glazing for directional thermal energy management. *Mater. Des.* **188**, 108407 (2020).

[S4] Wu, D., Meng, Y. & Liu, C. Design of transparent metasurfaces based on asymmetric nanostructures for directional and selective absorption. *Materials (Basel).* **13**, 3751 (2020).

[S5] Günay, A. A. *et al.* Optically Transparent Thermally Insulating Silica Aerogels for Solar Thermal Insulation. *ACS Appl. Mater. Interfaces* **10**, 12603–12611 (2018).
